# Dietary Patterns of 479 Indonesian Adults and Their Associations with Sodium and Potassium Intakes Estimated by Two 24-h Urine Collections

**DOI:** 10.3390/nu14142905

**Published:** 2022-07-15

**Authors:** Dianis Wulan Sari, Maiko Noguchi-Watanabe, Satoshi Sasaki, Noriko Yamamoto-Mitani

**Affiliations:** 1 Department of Gerontological Home Care and Long-term Care Nursing, Division of Health Sciences & Nursing, Graduate School of Medicine, The University of Tokyo, 7-3-1 Hongo, Bunkyo-ku, Tokyo 113-0033, Japan; noguchimaiko.chn@tmd.ac.jp; 2 Faculty of Nursing, Universitas Airlangga, Jln. Mulyorejo, Surabaya, East Java 60115, Indonesia; 3 Department of Home Care Nursing Science, School of Health Care Sciences, Tokyo Medical and Dental University, 1-5-45 Yushima, Bunkyo-ku, Tokyo 113-8510, Japan; 4 Department of Social and Preventive Epidemiology, School of Public Health, Graduate School of Medicine, The University of Tokyo, 7-3-1 Hongo, Bunkyo-ku, Tokyo 113-0033, Japan; stssasak@m.u-tokyo.ac.jp

**Keywords:** diet, dietary pattern, intake, sodium, potassium, 24-h urine collection

## Abstract

The excess sodium (Na) intake and insufficient potassium (K) intake are frequently observed all over the world, including Indonesia. This study explored the dietary patterns of Indonesian people and evaluated their associations with Na and K intakes. Na and K intakes were assessed by repeated 24-h urine collection. The dietary patterns of the previous month were extracted by factor analysis using the Indonesian Food Frequency Questionnaire. The participants were community-dwelling Indonesian men and women (n = 479) aged 20 years and over. We identified four dietary patterns in each sex. After controlling for confounding factors, the high quantile of ‘Noodle, oil, and salty sea products’ pattern was associated with the high Na intake in both men and women (*p* = 0.02 and <0.001, respectively). The ‘Meat, vegetable, oil, and fruit’ pattern statistically significantly contributed to the high K intake in men (*p* = 0.04), but not in women (*p* = 0.26). The ‘Vegetable, non-oil, and milk’ pattern in men and ‘Meat, vegetable, and fruit’ pattern in women were associated with low Na:K ratios (*p* = 0.03 and 0.01, respectively). Neither ‘bread’ nor ‘fish’ appeared as a major determinant of any dietary patterns in this population. The ‘Noodle, oil, and salty sea products’ pattern should be avoided to reduce sodium intake.

## 1. Introduction

A high sodium (Na) intake and low potassium (K) intake are common serious issues all over the world [1,2,3]. An excess Na intake induces arterial stiffness [4], and insufficient K affects the total body fluid volume, electrolyte balance, and normal cell function [5]. Therefore, a high Na intake and low K intake are independent risk factors that increase the probability of stroke and heart disease [4,6,7]. An Indonesian study showed that more than 80% of the participants were estimated to consume more Na than the recommended value using two 24-h urine excretions [8]. Moreover, there were no participants whose K intake met the recommended value. Dietary sources of Na and K vary widely across populations and cultures [9,10]. To understand Na and K intakes from diet among Indonesian people, a study to explore the variety of Na and K contents of Indonesian food is needed.

Many studies showed that high levels of Na are found in processed meat, fishery products, and other industrially processed food [11,12,13]. However, individuals do not consume each food separately; food is consumed in a structured way, composed of a variety of foods [14]. The influence of a food component can differ depending on the food it is derived from due to interactions between food components or physical characteristics of foods [15]. Therefore, the health effects of dietary patterns may be greater as compared to individual foods or nutrients.

A dietary pattern is defined as a set of foods consumed by a specific population, which gives information on habitual intake [16,17]. It is used in nutritional epidemiology due to the practical advantage of advising people as a dietary pattern, rather than advice based on the nutrient [18,19]. Grouping foods into dietary patterns can reflect the actual food consumption and show associated dietary patterns of high Na and K intakes [9,20]. Therefore, a dietary pattern with low Na and high K intakes can be applied directly and effectively in a nutritional programme to prevent hypertension and reduce heart disease risk.

However, only a few studies are available to confirm the associations between dietary patterns with Na and K intakes using precise samples and methods (in Japan and the UK) [9,10]. Particularly, for the Indonesian diet, no study has explored the sources of Na and K intakes in dietary patterns precisely. Thus, a study of Na and K sources in the Indonesian diet is important to customise a salt reduction programme.

The aims of this study were to explore the current dietary pattern of Indonesian people and to evaluate the association between Indonesian dietary patterns and Na and K intakes measured by repeated 24-h urine collection.

## 2. Materials and Methods

### 2.1. Study Participants and Study Area

Details of the study design, area, and procedure have been described elsewhere [8]. Briefly, the study was based on the data of a cross-sectional survey among community-dwelling people in an Indonesian city: men and women aged 20 years and over. Exclusion criteria were: (1) unable to provide informed consent, (2) difficulty in memorising or communicating, (3) under diet therapy prescribed by a doctor or dietitian at the time of the study or within 1 year before the study, (4) fasting, (5) severe diseases (i.e., kidney failure, total paralysis, and liver disease), and other conditions which made the 24-h urine collection difficult. The exclusion criteria were to ensure accurate dietary intake data and the 24-h urine collection completeness. The participants were included with randomised sampling using a computer random number generator based on the residents’ ID card numbers. To detect an approximately 1 g reduction in salt intake over time, using 24-h urinary Na excretion, with a standard deviation of 75 mmol/day (alpha = 0.05, power = 0.80), minimum sample was 120 individuals per group [21]. We invited 528 for 132 candidates per group (young men, young women, old men, and old women). The areas of data collection were Nganjuk City, a city in the East Java province of Indonesia. Nganjuk City is representative of sub-urban area, flat and non-coastal city in Indonesia. Data collection was performed in participants’ homes. 

### 2.2. Measurement Schedule

The data of each participant were collected over 4 days. The first day after the participant agreed to participate, all measurements were performed, the steps for collecting urine were further explained, and all materials were provided for 24-h urine collection. The schedule of urine collection was confirmed by both the researcher and the participant. The data collection was conducted from November 2018 to September 2019.

### 2.3. Dietary Intake

Dietary behaviour during the previous month was assessed using the Food Frequency Questionnaire (FFQ) [22]. The FFQ is based on the population food reference in Jakarta, where we expected a close dietary behaviour to that of this study population. The researchers conducted a validity analysis of this FFQ compared with 3-day 24-h food recall (FR) [23]. This method was confirmed in previous studies performed to examine the relative validity of FFQ [24,25]. Participants were asked to specify, on average, how often and how much they consumed each food over the previous month.

The FFQ consisted of questions on “usual portion” and “frequency of consumption” for each food listed. A “usual portion” was answered by the number. It was responded to by referring to the standard portion indicated in FFQ. For “frequency of consumption”, the most appropriate category was chosen from the following four categories: “1 day (times) 1–6”, “1 week (times) 1–6”, “1 month (times) 1–3”, and “Never”. For example, a participant usually eats quail eggs three times per week, they wrote (✓) on category “1 week (times) 1–6”. 

The original FFQ has 99 food items. However, participants can add new food items that they consume, which are not listed. Therefore, some participants added new food items in their responses. Finally, 125 total food items were included in this study. The FFQ has 17 categories: carbohydrate, roots and tubers, meat and poultry, fish and seafood, eggs, soybean, oil and fats, vegetable A (low energy), vegetable B (medium energy), vegetable C (high energy), mixed vegetable, fruits, snack, dessert, milk and milk products, tea–coffee and coffee milk, and sweetened beverages. For the analysis, we categorised the FFQ food items into 22 groups based on the nutrient profiles, process of cooking, and culinary usage of the foods.

### 2.4. 24-h Urine Collection

Na and K intakes were estimated from urine excretion using 2 consecutive days of 24-h urine collection. Details of the procedure for 24-h urine collection were given elsewhere [8]. The protocol was based on the WHO/PAHO report and based on a previous study conducted in Japan [21,26]. Briefly, we prepared documents for a protocol to collect 24-h urine that included the start time, finish time, and loss of urine (frequency and estimation of volume). Participants were asked to start urine collection in the morning (5.00 a.m.) and to finish the next day at the same time (5.00 a.m.). Participants could start collecting between 4:45–5:30 a.m. However, they were required to change the finish time accordingly. All the urine during 24 h had to be collected except the first urine at the start time. A participant received 4 bottles/day with 1-L capacity for each bottle. The research team collected the urine bottles and self-reported documents from participants’ homes daily.

The Prodia Medical Laboratory picked up the total urine sample. The volume of 24-h urine was adjusted by the medical laboratory based on the total urine collected and the participant’s self-report estimating the missing urine volume. This method was validated using the para-aminobenzoic acid (PABA) check method, as previously described [27]. The urine biomarker of creatinine urine (Cr) was analysed at Prodia Medical Laboratory, using CREP2 creatinine plus ver.2 (in vitro test [enzyme test]) for quantitative determination of creatinine concentration in urine on the Cobas c 111 system (Roche Diagnostic, Indianapolis, IN, USA). To examine the biomarkers of Na and K in urine, this medical laboratory company used the ISE indirect Na-K-Cl for Gen.2. The ISE module of the Roche/Hitachi Cobas c system (Roche Diagnostic, Indianapolis, IN, USA) was employed for quantitative determination of Na and K in urine using ion-selective electrodes. Excretion of Na and K and Na:K ratio were calculated as:

Total 24-h (Na and K) excretion (mmol/d) = adjusted volume of 24-h urine (mL/d) × concentration (mmol/mL).

Na:K was calculated by dividing excreted Na (mg/d) by K (mg/d).

### 2.5. Other Measurements

We evaluated participants’ characteristics based on the STEPS instrument, including age, sex, education level, and tobacco use [28]. Additionally, we measured daily activities using the Barthel Index (only in the old adult population) [29], cognitive status using the Mini-Cog© (only for old people) [30], and Physical Activity Level (PAL) from International Physical Activity Questionnaire (IPAQ) [31,32]. Further, physical examinations were conducted for blood pressure, body weight, and height. Clinical blood pressure technique [33,34], which requires the usage of electronic monitors thrice (Omron HEM-7130 with cuff Omron HEM-CR24 by Omron Healthcare CO. Ltd., Muko, Japan), was employed for measuring blood pressure. The protocol of blood pressure was based on the British Hypertension Society (BHS) guideline and WHO/PAHO regional expert group for cardiovascular disease [21,35,36]. Body weight (kg) was measured using a portable digital scale (Omron HN-286 by Omron Healthcare CO. Ltd., Muko, Japan) in units of 100 g. It had a capacity of 180 kg. Body height (cm) was measured using a portable stadiometer (with a capacity of 2 m) to the nearest 0.1 cm. The protocol of body weight and height examination was according to the instruction of STEPS [28]. All measurements were conducted based on a standardised protocol by well-trained researchers.

### 2.6. Data Analysis

Diet and urine excretion were analysed for men and women separately. A previous study reported differences in dietary patterns between sexes [37]. For dietary pattern analysis, we included 479 participants (240 men and 239 women) whose 24-h urine collections were considered complete at least once (assessed by calculating the ratio of observed to expected creatinine excretion according to the equation of Joossens et al.), and completeness of 24-h urine collection was based on the volumes of urine and self-reports by participants [27,38,39]. The mean was used when both samples were considered complete, while only one sample was used if the other was incomplete. We also excluded those from the study who were receiving dietary counseling from a doctor or dietitian at the beginning of data collection.

The FFQ has a value of the amount of food consumption and the frequency of consumption in categories. We converted this into frequency per month by multiplication with 0 (for ‘never’), 2 (for ‘1–3 times/month’), 12 (for ‘1–6 times/week’), and 84 (for ‘1–6 times/day’). Those numbers were assumed from the median of each category (e.g., for eating 1–3 times/month, the number used was 2, on the assumption that for 1 month the median of frequency for eating was 2 times; for eating 1–6 times/week, the number used was 12, on the assumption that for 1 month the median of frequency for eating was 12 times; and for eating 1–6 times/day, the number used was 84, on the assumption that for 1 month the median of frequency for eating was 84 times). We multiplied the usual portion and the frequency of consumption per month [times/month] to calculate the intake of each food item [usual portion] X [frequency of consumption/month]. Finally, the value of consumption of each food item was used for analysis [portion(s)/day].

Before conducting factor analysis, we needed an adjustment for energy intake. We adjusted energy using the body weight (kg) method instead of normal energy adjustment using energy intake. This study used a simple dietary questionnaire (FFQ), whereas total energy intake is usually measured using details of serving sizes that this FFQ does not assess. Hence, we used body weight to adjust for energy intake among participants. Previous studies have confirmed the association between energy intake and change in body weight [40,41]. Energy adjusted was calculated using the FFQ value [portion(s)/month] divided by body weight [kg]. Therefore, the final unit of FFQ value was portion(s)/month/[kg body weight].

Factor analysis was used to derive dietary patterns based on the 22 food groups of the FFQ. Twenty-two predefined food groups were evaluated using the Factor function of the SPSS software. Factor analysis (principal component estimation method) was conducted based on food group intakes. We applied the data to the factor analysis and verified it using the Kaiser–Meyer–Olkin (KMO) test and the Bartlett sphericity test. The data were acceptable if the values were over 0.50 and *p* < 0.05 on the KMO and the Bartlett sphericity test, respectively [42]. The selection of the number of factors was based on the latent root criteria, with eigenvalues > 1.5, the scree plot test, and the interpretability of the combination of food groups on the factors. We confirmed that no variable showed a commonality having a value ≤0.10. Subsequently, the factors were rotated by orthogonal transformation (Varimax rotation) to achieve a simpler structure with greater interpretability.

A factor solution with four factors was found to be reasonable and meaningful for both men and women. The food groups having factor loadings > 0.40 were considered as representatives of the pattern. Evaluation of the dietary patterns using factor analysis was based on the nomenclature used in the previous studies [9,20,22,43,44,45,46]. The proportion of variance of each factor was calculated by dividing the sum of the square of the respective factor loading by the number of variables. The factor scores of each dietary pattern for each participant were calculated by summing the intakes of food groups weighted by their factor score loadings. 

The factor score for each dietary pattern was categorised into quantiles (Q1 to Q4), and participants’ characteristics were compared among these categories. We used the Mantel–Haenszel chi-squared test for categorical variables and a linear trend for continuous variables. A linear regression analysis was performed to assess trend associations, using factor scores of dietary patterns (continuous) as independent variables. To explore which dietary patterns had high Na and K excretions and high Na:K ratios, we analysed the associations among quantiles (Q1 to Q4) of factor scores with the values of urinary Na and K excretions and Na:K ratios. The multivariate-adjusted means of Na and K excretions and Na:K ratios in each quantile were calculated by adjusting for age (Model 1). We further adjusted for education (low, middle, and high level), smoking (yes and no) (men only), and PAL (metabolic equivalent of task [MET], a total of the MET x h) in Model 2. Body weight was not used in the model because it was added for adjustment of energy intake. Trends of association in factor score quantiles were assessed by a linear regression model which included Na and K excretion or Na:K ratio as a dependent variable, and factor scores of the dietary pattern (continuous), age, education, smoking, and PAL as independent variables. The adjusted factors in the model were considered based on significance of the factors in Table 1 and Table 2, as well as from a previous study that mentioned the effects of these factors on urine excretion or food consumption. Finally, the characteristic Na and K intakes in each dietary pattern could be observed from the mean value of Na and K excretions on the highest quantile of each dietary pattern. All analyses were performed using SPSS version 26 (IBM, Armonk, NY, USA). *p*-values < 0.05 were considered statistically significant.

## 3. Results

### 3.1. Characteristics of Participants

Participants’ characteristics are shown in Table 1 and Table 2. In this study, we recruited 528 people and 22 people did not participate. The completed questionnaires were obtained from 506 people. Among them, 22 people failed to collect urine. Therefore, 484 people completed the questionnaire and urine collection. Finally, 240 men and 239 women (479 people) with complete 24-h urine collections were included in the analysis. For men, the mean age and BMI were 57.0 years and 20.9, respectively; for women, the mean age and BMI were 56.0 years and 23.7, respectively. 

### 3.2. Factor Analysis of Dietary Pattern

Factor analysis was conducted for the 22 food groups (Supplementary Table S1). The KMO values were >0.5 and Bartlett’s test had a *p* value < 0.001 for all food groups; this indicated that all food groups could be included in the factor analysis. Four factors of the dietary pattern resulted in each sex. The eigenvalues were higher than 1.5. Factors were labelled based on factor loadings > 0.4. There were no dietary patterns that had the highest loadings for more than one factor. The percentage of total cumulated variance was 38.5% (men) and 40.7% (women) (Supplementary Table S2). The men’s pattern 1 (M1), ‘Meat, vegetable, oil, and fruit’, had high positive loadings on poultry, red meats, vegetables with additional elements contained (peanut sauce, oil, or coconut milk/salted vegetables), fruit, fried snacks, and desserts. The men’s pattern 2 (M2), ‘Staples, oil, and sweet’, had high positive loading on staples, fried soybean products, and tea/coffee/soft drinks (mostly consumed sweet). The men’s pattern 3 (M3), ‘Noodle, oil, and salty sea products’, had high factor loading on staples with fat/oil, noodle, root/tubers (mostly fried), and salty sea products. The men’s pattern 4 (M4), ‘Vegetable, non-oil, and milk’, had high positive loading on staples, non-fried soybean products, raw vegetables, and milk. For women, the women pattern 1 (W1), ‘Meat, vegetable, and fruit’, had high loading on poultry, red meat, vegetables, and fruits. The women pattern 2 (W2), ‘Staples, oil, and sweet’, had high loading on staples, root/tubers (mostly fried), fried soybean products, desserts, milk, and tea/coffee/soft drinks (mostly consumed sweet). W3, ‘Noodle, oil, and salty sea products’, had high factor loading on noodles, processed meat products, salty sea products, and fried snacks. The women pattern 4 (W4), ‘Composite and non-oil’, had high factor loading on non-fried soybean products, composite foods, and non-fried snacks.

### 3.3. Dietary Pattern and Participant’s Characteristics

The analysis between dietary pattern and participant’s characteristics is shown in Table 1 (men) and Table 2 (women). Participants were divided into quantiles (Q) based on the factor score of each dietary pattern. The lowest quantile was Q1, while the highest was Q4. We measured significant differences among the quantiles (Q1 and Q4). In men, the M1 ‘Meat, vegetable, oil, and fruit’ pattern had positive associations with a young age, high educational level, and smoking. Conversely, the M2 ‘Staples, oil, and sweet’ pattern showed associations with BMI, PAL, and smoking. The M3 ‘Noodle, oil, and salty sea products’ pattern had associations with BMI and PAL. Further, the M4 ‘Vegetable, non-oil, and milk’ pattern had positive associations with old age, low education, BMI, and PAL. In women, the W1 ‘Meat, vegetable, and fruit’ pattern had a positive association with a young age. The W2 ‘Staples, oil, and sweet’ pattern had positive associations with old age and PAL. However, the W3 ‘Noodle, oil, and salty sea products’ pattern had no significant associations with a participant’s characteristics. Moreover, it was prevalent among young people. Further, the W4 ‘Composite and non-oil’ pattern had a positive association with old age.

### 3.4. The Multivariate-Adjusted Means for Urine Excretion throughout the Quantiles of Dietary Patterns

Table 3 (men) and Table 4 (women) summarise the multivariate-adjusted means for Na and K excretions and the Na:K ratios throughout the quantiles of all the dietary patterns. In men (Table 3), after controlling for confounding factors, higher factor scores for the M1 ‘Meat, vegetable, oil, and fruit’ pattern were significantly associated with higher Na and K excretions (*p* for trend = 0.02 and 0.04, respectively). Thus, Na and K excretions in the highest quantile (Q4) of the M1 ‘Meat, vegetable, oil, and fruit’ pattern were higher than that in the lowest quantile (Q1). The M3 ‘Noodle, oil, and salty sea products’ pattern was significantly associated with higher Na and K excretions (*p* for trend 0.02 and <0.001, respectively). Thus, Na and K excretions in the highest quantile (Q4) of the M3 ‘Noodle, oil, and salty sea products’ pattern were higher compared to the lowest quantile (Q1).

For women (Table 4), higher factor scores for the W1 ‘Meat, vegetable, and fruit’ pattern were significantly associated with lower Na:K ratios (P for trend 0.01). The mean Na:K ratio in the highest quantile (Q4) of the W1 ‘Meat, vegetable, and fruit’ pattern was 0.9 times lower than those in the lowest quantile (Q1). The mean K excretion of the highest quantile (Q4) in the W1 ‘Meat, vegetable, and fruit’ pattern was the highest among all the patterns, but it was not statistically different. Further, the W3 ‘Noodle, oil, and salty sea products’ pattern was significantly associated with high Na excretion and a high Na:K ratio (*p* for trend < 0.001 and 0.002, respectively). Thus, Na excretions and the Na:K ratios among participants in the highest quantile (Q4) of the W3 ‘Noodle, oil, and salty sea products’ pattern were higher compared to the lowest quantile.

## 4. Discussion

This study explored the dietary patterns of Indonesian people and evaluated the association between Indonesian dietary patterns and Na and K intakes measured by repeated 24-h urine collection, which is considered the gold standard [47]. To the best of our knowledge, this is the first study to investigate the associations of dietary patterns with Na and K intakes using precise sample size and gold standard, among Indonesia’s population. 

We found dietary pattern differences between young and old populations. Young adults had high factor scores on the M1 ‘Meat, vegetable, oil, desert, and fruit’ pattern, W1 ‘Meat, vegetable, and fruit’ pattern, and W3 ‘Noodle and salty sea products’ pattern, while old people had high factor scores on the M4 ‘Vegetable, non-oil, and milk’ and W4 ‘Composite and non-oil’ patterns. This might explain a young adult’s high Na intake [8]. A previous study reported that sources of salt differed among generations [48]. Young and old generations have different preferences for dietary patterns. This shift in the dietary pattern is commonly called nutrition transition [49,50]. This transition is prevalent in developing countries [51]. Major demographic and socioeconomic changes of a country paralleled with changes in dietary patterns. The dietary pattern was changed with the increase of over-Na and poor-K foods [52]. The young generation tends to consume processed foods [53]. Therefore, we should consider different dietary patterns among generations to develop a salt reduction programme. 

The high factor scores of the M3 ‘Noodle, oil, and salty sea products’ and W3 ‘Noodle, oil, and salty sea products’ patterns were significantly associated with a high urinary Na excretion. High Na in these patterns might be due to a high intake of noodles with excess salty seasoning, fried food, processed meat, and salty sea products (salted fish, anchovies, and fried food). The characteristics of this pattern were comparable with those of traditional Indonesian foods, which are preserved by marinating and frying. Salt is added to most Indonesian fried foods while cooking. This pattern is similar to the dietary pattern in a previous study among normal and obese women in Indonesia, labelled the ‘less healthy’ pattern. The ‘less healthy’ pattern has a high loading of fried food and animal ingredients [22]. This result is also similar to that of a Japanese study, which showed that the noodle pattern is associated with high Na intake [9]. Noodles and processed meat are processed foods, and it is well known that processed foods are associated with a high salt intake [54,55,56]. Therefore, this dietary pattern contains high levels of Na. 

Furthermore, the high factor scores of the M1 ‘Meat, vegetable, oil, and fruit’ and W1 ‘Meat, vegetable, and fruit’ patterns were associated with high urinary K excretion. Fruits and vegetables are important sources of K, along with milk, beans, and nuts [57,58,59]. However, meat has been found to have a considerable impact on Na intake, similar to fried food [60]. Thus, the high factor loading of meat and oily food could increase the Na intake. Further, the load of oily food is high in dietary patterns among men; therefore, the M1 ‘Meat, vegetable, oil, and fruit’ pattern was also significantly associated with a high Na intake. This pattern was similar to the dietary pattern labelled the ‘Western’ pattern in a Japanese study where the high factor loading of vegetables and fruit was found to be higher in women [61]. This study confirmed that the ‘Meat, vegetable, and fruit’ pattern has a high K intake. However, the loading of ‘oil’ and ‘dessert’ in dietary patterns of men was related to sex. Therefore, the ‘Meat, vegetable, and fruit’ pattern should be recommended carefully to Indonesian people.

The M4 ‘Vegetable, non-oil, and milk’ and W1 ‘Meat, vegetable, and fruit’ patterns were associated with low Na:K ratios. The low Na:K ratio is influenced by the balance of Na and K excretions. The M4 ‘Vegetable, non-oil, and milk’ pattern is associated with a low Na intake. The loading of vegetables and fruits in the M4 ‘Vegetable, non-oil, and milk’ and W1 ‘Meat, vegetable, and fruit’ patterns was related to high K and a low Na:K ratio in this study. This result is similar to that of a previous study, wherein vegetables and fruits were associated with high K and a low Na:K ratio in the urine [57]. Reducing the urine Na:K ratio is important to decrease blood pressure and the risk of heart disease [62,63]. There is no strict cutoff value recommended for the urinary Na:K ratio for a specific population. However, a Na:K ratio < 1 might be a useful indicator of adherence to the recommended values of Na and K intakes [64,65]. In this study, the Na:K ratio was >1 among men and women. Therefore, reducing the Na:K ratio is challenging in this study population. 

The results of the dietary patterns in this study differed from those of some previous studies in Western countries [10,66] and slightly differed from the results of a study in Japan [9]. In Western countries, a high Na intake was associated with the ‘bread and meat product’ pattern, whereas the ‘fish and vegetable’ pattern showed no association [10,66]. In Japan, a high Na intake was associated with the high loading of ‘fish and vegetable’ and ‘noodle’ patterns [9]. Likewise, in the current study, the highest Na intake was present in the M3 ‘Noodle, oil, and salty sea products’ and W3 ‘Noodle, oil, and salty sea products’ patterns. The traditional food type in each country differs; therefore, the contribution of each food group to the total intake might also differ. Generally, Indonesian people do not consume bread frequently, and most fish consumed is inland (white-water) fish. These may be the reasons why the ‘Bread’ and ‘Fish’ patterns in this study did not contribute to factor loading.

This result highlights the fact that the salt reduction programme of each country should be developed carefully. Each country must figure out the sources of salt in their dietary patterns in order to implement public campaigns or salt reduction programmes. For example, a salt reduction programme in the UK targeted food categories that contribute most to the population’s salt intake (i.e., bread and processed food products) by means of a public campaign and by the regulation of the food industry since 2006, which was effective [67,68]. Targeting the dietary pattern in each region and population is necessary to ensure the success of a salt reduction programme. 

In the future, based on the results of this study, it would be recommended that a nutrition programme focused on reducing ‘Noodle, oil, and salty sea products’ is needed for Indonesians. An implementation study about nutrition programmes based on dietary patterns should be commenced first at schools to evaluate the effect of such public campaigns for decreasing salt intake. Since eating habits are formed during childhood or adolescence, an educational programme for salt reduction should be initiated at an earlier age and continued until a later age. Such health education can be conducted in an Integrated Guidance Post (Posbindu and Posyandu) and primary schools.

This study has several limitations. First, we used 24-h urine to measure the Na and K excretion. Briefly, the standardisation of the 24-h urine collection was difficult because this study was conducted in a tropical country, with high equatorial temperatures almost everywhere. However, we collected the temperature and humidity data during urine collection. Then, we compared the results of urine from higher and lower temperatures and humidities. No significant differences were found [8]. Second, the factor analysis itself has limitations as the process emerged from arbitrary decisions. This procedure might have affected the results or their interpretations. We tried to stabilise the factor analysis by ensuring the ratio of variables and the number of samples were precise. Moreover, we performed dietary pattern analysis using procedures similar to those of a previous study for objective decision making, such as selecting the eigenvalues, scree plots, and interpretability. We had many discussions with panel experts of nutrition epidemiology to confirm and to interpret the results of the factor analysis. Third, the calculation method of the dietary patterns used in the present study has a severe shortcoming. As both the information on the weight for the standard portions for the foods listed in the FFQ and a food composition table with energy were lacking, we, therefore, used “the number of portions”, instead of “grams” or “energy”, for this calculation. This might have given incorrect importance between foods, for example, one consumption of chicken egg and one consumption of quail egg were dealt with the same weighting.

## 5. Conclusions

In conclusion, there were four types of dietary patterns among Indonesian men and women. Among participants of both sexes, the high quantile of the M3 ‘Noodle, oil, and salty sea products’ and W3 ‘Noodle, oil, and salty sea products’ patterns was associated with the highest Na intake. The M1 ‘Meat, vegetable, oil, and fruit’ and W1 ‘Meat, vegetable, and fruit’ patterns contributed to the highest K intake. Finally, the M4 ‘Vegetable, non-oil pattern and milk’ pattern in men and W1 ‘Meat, vegetable, and fruit’ pattern in women had low Na:K ratios.

Based on the above findings, the consumption of noodles, fried foods, processed meat, and salty dried fish should be reduced. It should be noted that salt intake was higher among the younger people, which will result in the development of hypertension in their later life. Since food habits are formed during childhood or adolescence, an educational program that aims to reduce salt intake, including dietary patterns, should be initiated in primary school and continued in secondary schools. An appropriate educational programme for children and adolescents will be beneficial to reducing salt intake and consuming more vegetables and fruits rich in potassium.

## Figures and Tables

**Table 1:** Participants’ characteristics for the lowest (Q1) and highest (Q4) for men *n* = 240 *.

	All (*n* = 240)		M1 ‘Meat, Vegetable, Oil, and Fruit’					M2 ‘Staples, Oil, and Sweet’					M3 ‘Noodle, Oil, and Salty Sea Products’					M4 ‘Vegetable, Non-Oil, and Milk’				
			Q1 (*n* = 60)		Q4 (*n* = 60)		*p* Value ^†^	Q1 (*n* = 60)		Q4 (*n* = 60)		*p Value* ^†^	Q1 (*n* = 60)		Q4 (*n* = 60)		*p Value* ^†^	Q1 (*n* = 60)		Q4 (*n* = 60)		*p Value* ^†^
Mean (SD) or *n* (%)			Mean (SD) or *n* (%)					Mean (SD) or *n* (%)					Mean (SD) or *n* (%)					Mean (SD) or *n* (%)				
Age (years)	57.0	14.8	64.7	12.2	52.9	13.9	<0.001	55.4	16.0	58.8	14.9	0.63	58.7	16.3	60.2	13.4	0.05	51.7	12.3	64.4	14.9	<0.001
Age group																						
20–59 years	127	52.9	18	30.0	40	66.7	<0.001	33	55.0	28	46.7	0.39	28	46.7	25	41.7	0.60	46	76.7	20	33.3	<0.001
60–99 years	113	47.1	42	70.0	20	33.3		27	45.0	32	53.3		32	53.3	35	58.3		14	23.3	40	66.7	
Body height (cm)	159.8	5.9	159.1	6.0	160.5	4.8	0.46	161.9	5.8	156.8	5.1	<0.001	160.0	6.2	159.3	5.4	0.49	160.8	5.2	157.7	7.1	0.02
Body weight (kg)	53.5	10.4	53.2	10.8	52.3	9.6	0.60	53.2	10.8	52.3	9.6	<0.001	61.7	11.5	44.4	5.6	0.006	54.5	11.9	49.8	8.1	<0.001
BMI (kg/m^2^)	20.9	3.7	20.9	3.7	20.3	3.3	0.42	23.6	4.5	18.1	2.3	<0.001	21.2	4.1	19.6	2.9	0.010	22.8	3.7	18.6	2.9	<0.001
SBP (mmHg)	139.9	23.9	148.1	28.1	132.4	19.9	0.002	139.4	22.3	135.3	22.6	0.30	140.9	25.0	140.1	26.0	0.73	144.9	23.8	135.3	26.0	0.13
DBP (mmHg)	87.4	11.8	88.9	11.9	85.4	10.4	0.39	88.7	12.4	84.7	11.8	0.20	88.4	12.9	86.4	11.1	0.56	91.9	12.1	83.0	11.8	<0.001
PAL (MET·h)	67.2	56.4	54.2	53.7	76.6	59.4	0.14	52.0	50.8	83.3	60.0	0.010	49.1	51.5	75.4	61.7	0.03	91.9	12.1	83.0	11.8	<0.001
Education level ^‡^																						
Low	54	22.5	17	28.3	11	18.3	<0.001	11	18.3	18	30.0	<0.001	11	18.3	20	33.3	0.06	9	15.0	19	31.7	0.03
Middle	124	51.7	40	66.7	26	43.3		21	35.0	36	60.0		31	51.7	30	50.0		36	60.0	28	46.7	
High	62	25.8	3	5.0	23	38.3		28	46.7	6	10.0		18	30.0	10	16.7		15	25.0	13	21.7	
Smoking																						
Yes	144	60.0	28	46.7	42	70	0.010	24	40.0	45	75.0	<0.001	28	46.7	38	63.3	0.06	42	70.0	28	46.7	0.010
No	96	40.0	32	53.3	18	30		36	60.0	15	25.0		32	53.3	22	36.7		18	30.0	32	53.3	

Q, quantile; SD, standard deviation; BMI, body mass index; SBP, systolic blood pressure; DBP, diastolic blood pressure; PAL, physical activity level; MET, metabolic equivalent of task. * The factors were standardized by continuous variables, and each participant had a score for each factor. ^‡^ Education level: low (no school—not completed primary school), middle (completed primary—secondary school), high (completed high school—more). ^†^ A linear trend test and the Mantel–Haenszel χ^2^ test were used for continuous and categorical variables, respectively.

**Table 2:** Participant’s characteristics for the lowest (Q1) and highest (Q4) for women *n* = 239 *.

	All (*n* = 239)		W1 ‘Meat, Vegetable, and Fruit’					W2 ‘Staples, Oil, and Sweet’					W3 ‘Noodle, Oil, and Salty Sea Products’					W4 ‘Composite and Non-Oil’				
			Q1 (*n* = 60)		Q4 (*n* = 59)		*p Value* ^†^	Q1 (*n* = 60)		Q4 (*n* = 59)		*p Value* ^†^	Q1 (*n* = 60)		Q4 (*n* = 59)		*p Value* ^†^	Q1 (*n* = 59)		Q4 (*n* = 60)		*p Value* ^†^
	Mean (SD) or *n* (%)		Mean (SD) or *n* (%)					Mean (SD) or *n* (%)					Mean (SD) or *n* (%)					Mean (SD) or *n* (%)				
Age (years)	56.0	14.6	61.4	14.6	54.8	14.6	0.01	54.2	16.2	64.0	11.4	<0.001	58.8	13.3	53.3	15.4	0.24	48.2	12.9	65.6	13.0	<0.001
Age group																						
20–59 years	130	54.4	23	38.3	38	64.4	0.008	34	57.6	20	33.3	0.003	29	48.3	36	61.0	0.17	49	81.7	15	25.4	<0.001
60–99 years	109	45.6	37	61.7	21	35.6		25	42.4	40	66.7		31	51.7	23	39.0		11	18.3	44	74.6	
Body height (cm)	151.1	6.0	151.6	8.2	150.5	5.0	0.50	153.2	5.1	148.8	8.0	0.001	150.0	5.7	151.4	5.4	0.44	152.0	8.0	150.3	5.2	0.43
Body weight (kg)	54.2	10.7	54.8	11.7	51.3	9.1	0.10	61.5	10.4	45.1	8.3	<0.001	53.9	11.5	52.1	10.5	0.11	55.9	9.3	51.7	11.7	0.10
BMI (kg/m^2^)	23.7	4.2	23.8	4.6	22.6	3.8	0.11	26.1	3.8	20.4	3.7	<0.001	23.9	4.4	22.7	4.1	0.06	24.2	3.8	22.8	4.5	0.09
SBP (mmHg)	138.7	25.5	145.1	25.1	136.8	26.3	0.04	139.2	24.4	141.3	23.8	0.77	139.9	26.2	133.6	25.9	0.15	130.0	23.3	142.9	23.0	0.02
DBP (mmHg)	87.3	10.7	90.0	11.2	85.0	9.9	0.03	88.3	10.5	86.3	9.6	0.80	87.3	11.1	86.4	9.3	0.32	85.6	9.5	86.8	10.0	0.26
PAL (MET·h)	75.2	56.7	69.6	53.7	81.3	60.9	0.35	54.7	45.3	78.3	55.8	0.01	64.2	58.1	79.0	56.1	0.20	72.5	53.8	66.9	53.0	0.48
Education level ^‡^																						
Low	80	33.5	24	40.0	19	32.2	0.04	14	23.7	36	60.0	<0.001	24	40.0	17	28.8	0.19	7	11.7	34	57.6	<0.001
Middle	107	44.8	30	50.0	23	39.0		23	39.0	20	33.3		27	45.0	25	42.4		38	63.3	18	30.5	
High	52	21.8	6	10.0	17	28.8		22	37.3	4	6.7		9	15.0	17	28.8		15	25.0	7	11.9	

Q, quantile; SD, standard deviation; BMI, body mass index; SBP, systolic blood pressure; DBP, diastolic blood pressure; PAL, physical activity level; MET, metabolic equivalent of task. * The factors were standardized by continuous variables, and each participant had a score for each factor. ^‡^ Education level: low (no school—not completed primary school), middle (completed primary—secondary school), high (completed high school—more). ^†^ A linear trend test and the Mantel–Haenszel χ2 test were used for continuous and categorical variables, respectively. Smoking habit was not calculated for female participants because nobody was smoking in this population.

**Table 3:** Multivariate-adjusted means and SE for urinary sodium and potassium excretion and sodium:potassium ratio by quintile of each dietary pattern for 240 Indonesian men.

	Quantiles Category of Dietary Patterns								
	Q1 (*n* = 60)		Q2 (*n* = 60)		Q3 (*n* = 60)		Q4 (*n* = 60)		
	Mean	SE	Mean	SE	Mean	SE	Mean	SE	*p Value* *
**M1 ‘Meat, vegetable, oil, and fruit’**									
Na excretion (mmol/d)									
Model 1	90.8	5.7	104.9	5.5	110.6	5.6	103.9	5.6	0.10
Model 2	91.4	5.8	104.7	5.5	110.6	5.6	103.4	5.7	0.02
K excretion (mmol/d)									
Model 1	21.9	1.2	25.3	1.1	25.5	1.2	27.0	1.2	0.02
Model 2	21.9	1.2	25.4	1.2	25.5	1.2	26.9	1.2	0.04
Na:K									
Model 1	6.8	0.4	6.7	0.3	6.9	0.3	6.0	0.3	0.27
Model 2	6.7	0.4	6.7	0.3	6.9	0.3	6.0	0.3	0.33
**M2 ‘Staples, oil, and sweet’**									
Na excretion (mmol/d)									
Model 1	112.3	5.5	109.9	5.5	90.5	5.5	97.6	5.5	0.02
Model 2	112.2	5.7	109.8	5.5	90.4	5.5	97.9	5.7	0.04
K excretion (mmol/d)									
Model 1	27.6	1.1	25.8	1.1	22.9	1.1	23.3	1.1	0.01
Model 2	28.2	1.2	26.0	1.1	22.7	1.1	22.8	1.2	0.003
Na:K									
Model 1	6.5	0.3	6.9	0.3	6.4	0.3	6.6	0.3	0.77
Model 2	6.3	0.4	6.8	0.3	6.4	0.3	6.8	0.4	0.72
**M3 ‘Noodle, oil, and salty sea products’**									
Na excretion (mmol/d)									
Model 1	89.4	5.5	104.4	5.5	108.5	5.6	107.9	5.6	0.05
Model 2	87.6	5.6	104.5	5.5	108.5	5.6	109.6	5.6	0.02
K excretion (mmol/d)									
Model 1	21.9	1.1	28.6	1.1	24.2	1.1	25.0	1.1	<0.001
Model 2	21.9	1.2	28.6	1.1	24.1	1.1	25.0	1.1	<0.001
Na:K									
Model 1	6.6	0.3	6.0	0.3	7.1	0.3	6.7	0.3	0.16
Model 2	6.5	0.3	6.0	0.3	7.1	0.3	6.8	0.3	0.13
**M4 ‘Vegetable, non-oil, and milk’**									
Na excretion (mmol/d)									
Model 1	116.8	5.5	112.6	5.4	97.8	5.4	83.1	5.6	<0.001
Model 2	117.2	5.5	111.7	5.5	98.4	5.4	83.0	5.6	<0.001
K excretion (mmol/d)									
Model 1	25.5	1.2	26.3	1.2	24.5	1.2	23.4	1.2	0.37
Model 2	25.5	1.2	26.4	1.2	24.6	1.2	23.2	1.2	0.30
Na:K									
Model 1	7.4	0.3	6.7	0.3	6.3	0.3	5.9	0.4	0.03
Model 2	7.4	0.3	6.6	0.3	6.3	0.3	6.0	0.4	0.03

Q, quantile; SE, standard error; mmol/d, mmol/day. * The trend analysis was conducted by a linear trend test. Model 1: adjusted for age. Model 2: further adjusted for education level (high, middle, and low level), smoking (yes and no), physical activity level (MET, total of metabolic equivalent of task·h).

**Table 4:** Multivariate-adjusted means and SE for urinary sodium and potassium excretion and sodium:potassium ratio by quintile of each dietary pattern for 239 Indonesian women.

	Quantiles Category of Dietary Patterns								
	Mean	SE	Mean	SE	Mean	SE	Mean	SE	*p Value* *
**W1 ‘Meat, vegetable, and fruit’**	**Q1 (*n* = 60)**		**Q2 (*n* = 60)**		**Q3 (*n* = 60)**		**Q4 (*n* = 59)**		
Na excretion (mmol/d)									
Model 1	105.8	4.4	98.2	4.3	97.4	4.3	100.3	4.4	0.54
Model 2	105.9	4.4	98.7	4.3	97.3	4.3	99.7	4.3	0.52
K excretion (mmol/d)									
Model 1	22.9	1.1	21.9	1.1	24.6	1.1	24.1	1.1	0.25
Model 2	23.0	1.1	21.9	1.0	24.7	1.0	24.0	1.1	0.26
Na:K									
Model 1	7.4	0.3	7.1	0.3	6.1	0.3	6.5	0.3	0.01
Model 2	7.4	0.3	7.1	0.3	6.1	0.3	6.5	0.3	0.01
**W2 ‘Staples, oil, and sweet’**	**Q1 (*n* = 59)**		**Q2 (*n* = 60)**		**Q3 (*n* = 60)**		**Q4 (*n* = 60)**		
Na excretion (mmol/d)									
Model 1	105.7	4.3	102.3	4.4	100.6	4.3	93.1	4.5	0.24
Model 2	104.9	4.5	102.0	4.3	100.6	4.3	94.2	4.5	0.42
K excretion (mmol/d)									
Model 1	25.9	1.1	23.2	1.1	22.8	1.0	21.7	1.1	0.05
Model 2	26.0	1.1	23.1	1.0	22.6	1.0	21.8	1.1	0.05
Na:K									
Model 1	6.3	0.3	6.9	0.3	7.2	0.3	6.7	0.3	0.24
Model 2	6.3	0.3	6.9	0.3	7.2	0.3	6.7	0.3	0.17
**W3 ‘Noodle, oil, and salty sea products’**	**Q1 (*n* = 60)**		**Q2 (*n* = 60)**		**Q3 (*n* = 60)**		**Q4 (*n* = 59)**		
Na excretion (mmol/d)									
Model 1	84.3	4.1	99.2	4.1	112.4	4.1	105.8	4.2	<0.001
Model 2	84.8	4.1	98.1	4.1	113.2	4.1	105.6	4.1	<0.001
K excretion (mmol/d)									
Model 1	21.3	1.1	24.6	1.1	23.9	1.1	23.8	1.1	0.13
Model 2	21.5	1.1	24.4	1.0	23.9	1.0	23.7	1.1	0.22
Na:K									
Model 1	6.2	0.3	6.3	0.3	7.4	0.3	7.3	0.3	0.003
Model 2	6.2	0.3	6.3	0.3	7.4	0.3	7.3	0.3	0.002
**W4 ‘Composite and non-oil’**	**Q1 (*n* = 59)**		**Q2 (*n* = 60)**		**Q3 (*n* = 60)**		**Q4 (*n* = 60)**		
Na excretion (mmol/d)									
Model 1	97.0	4.5	102.7	4.3	106.3	4.3	95.5	4.6	0.26
Model 2	97.1	4.5	102.2	4.3	106.3	4.3	95.9	4.6	0.29
K excretion (mmol/d)									
Model 1	22.9	1.1	24.2	1.1	24.3	1.1	22.2	1.1	0.44
Model 2	23.0	1.1	24.1	1.1	24.2	1.0	22.3	1.1	0.54
Na:K									
Model 1	6.8	0.3	6.6	0.3	7.1	0.3	6.8	0.3	0.70
Model 2	6.7	0.3	6.6	0.3	7.1	0.3	6.7	0.3	0.70

Q, quantile; SE, standard error; mmol/d, mmol/day. * The trend analysis was conducted by a linear trend test. Model 1: adjusted for age and body weight. Model 2: further adjusted for education level (high, middle, and low level), physical activity level (MET, total of metabolic equivalent of task·h).

## Data Availability

The data presented in this study are available on request from the corresponding author.

## Supplementary Materials

The following supporting information can be downloaded at: https://www.mdpi.com/article/10.3390/nu14142905/s1, Table S1: The food list used in the present study for dietary pattern analysis; Table S2: Factor-loading matrix for dietary patterns identified among Indonesian men (*n* = 240) and women (*n* = 239) *.
