# Supplementary material for: Dietary Patterns of 479 Indonesian Adults and Their Associations with Sodium and Potassium Intakes Estimated by Two 24-h Urine Collections
Source: Nutrients. 2022 Jul 15;14(14):2905. doi: 10.3390/nu14142905 (PMC9318011; doi:10.3390/nu14142905)
Supplement: Supplementary file 1 [file nutrients-14-02905-s001.zip › nutrients-1777836-supplementary.pdf]

Supplementary Table S1. The food list used in the present study for dietary pattern analysis

Food groups (n)	Food items
Staples (3)	Rice, corn rice
Staples with fat/oil (3)	Chicken porridge, rice (with coconut milk), fried rice
Noodle (7)	Soup rice vermicelli, *kwetiau* soup, noodles soup, fried noodles, fried rice vermicelli, chicken noodle, noodle *soto*
Root, tubers (2)	Cassava rice, root & tubers (boiled and fried)
Poultry (6)	Fried chicken/ chicken nugget, spicy chicken coconut milk, chicken satay, claw chicken, duck
Red meats (3)	Boiled cow's soft bone, stir fry cow's soft bone, *rendang*
Organ meats (5)	Fried gizzard, stir gizzard, fried liver, stir fry liver, fried chicken intestine
Processed meat product (2)	Grilled sausage, fried sausage
Fish and shellfish (6)	Fried fish/ spicy fried fish, steamed fish, stir fry squid, fried squid, fried shrimp/ spicy fried shrimp, grill fish
Salty sea products (3)	Anchovy with peanuts, wet salted fish, dries salted fish
Eggs (3)	Boiled eggs, quail eggs, fried eggs
Non-fried soybean product (2)	Boiled tofu, tofu/ tempeh steam
Fried soybean product (6)	Sweet fried tofu, fried tofu, sweet fried tempeh, fried tempeh, sweet and spicy fried tempeh, tofu with noodle (fried)
Composite foods (7)	Rice cake-peanut sauce (*ketoprak*), rice cake-coconut milk vegetable dish, rice cake with soy sauce (*lontong*), meat ball, chicken *soto*, brown beef soup (*rawon*), lamb curry soup (*gule*)
Raw vegetables (5)	Raw or boil vegetable A, B, C, sour mixed vegetable, mixed vegetable soup
Vegetables with additional contain (peanut sauce, oil or coconut milk) / salted vegetables (9)	Stir fry vegetable A and B, vegetable cooked with coconut milk, vegetable with coconut (*krawu)*, vegetable with peanut (*gado-gado/ pecel)*
Fruits (16)	All type of fruits
Non-fried snacks (7)	Biscuit crackers, corn, peanut, sticky rice, cakes, rice cake, bread
Fried snacks/ fried side dishes (15)	Fried *bakwan, cakwe, cireng,* doughnut, cassava chips, potato chips, egg stuffed pancake, fried fish ball, *pempek,* fried banana, *risol/ pastel*, potato/ corn fried dish, crackers frying, margarine, oil
Desert sweet with coconut milk (4)	Mug bean, rice flour, mixed ice, *kolak*
Milk and milk product (5)	Milk powder, white milk powder, chocolate milk powder, milo, condensed milk, instant cereal beverages, yogurt, UHT milk
Tea, coffee, soft drink (6)	All type tea (with/ without sugar), all type coffee (with/ without sugar); sweet, sweetened beverages

n : The number of questions in FFQ for each food group.

Supplementary Table S2. Factor-loading matrix for dietary patterns identified among Indonesian men (n=240) and women (n=239) *

**Food group**	**Men**				**Women**			
	M1'Meat, vegetable, oil, and fruit'	M2'Staples, oil, and sweet'	M3'Noodle, oil, and salty sea products'	M4'Vegetable, non-oil, and milk'	W1'Meat, vegetable, and fruit'	W2'Staples, oil, and sweet'	W3'Noodle, oil, and salty sea products'	W4'Composite and non-oil'
Staples	0.06	0.61	0.02	0.45	-0.10	0.59	0.15	0.16
Staples with fat/oil	-0.01	-0.22	0.78	-0.07	0.08	-0.02	0.06	0.13
Noodle[†]	0.11	0.00	0.64	-0.11	-0.11	-0.15	0.68	0.30
Root, tubers[†]	-0.13	0.20	0.68	0.03	-0.02	0.53	-0.16	0.00
Poultry	0.62	-0.22	0.17	-0.01	0.68	-0.29	0.28	-0.11
Red meats	0.48	0.00	-0.20	-0.26	0.60	-0.02	0.00	0.15
Organ meats	0.19	0.07	0.02	-0.04	0.05	0.07	0.36	-0.08
Processed meat product[†]	0.32	-0.26	-0.17	-0.03	0.03	-0.23	0.50	0.07
Fish and shellfish[‡]	0.34	-0.39	0.38	0.08	0.13	-0.30	0.14	-0.09
Salty sea products	0.03	0.34	0.44	-0.31	-0.13	0.28	0.53	0.02
Eggs	0.29	-0.08	-0.03	0.13	0.22	-0.14	0.30	0.21
Non-fried soybean product	-0.15	-0.01	-0.07	0.65	0.02	0.04	-0.28	0.71
Fried soybean product	0.01	0.58	-0.19	-0.16	0.13	0.50	0.24	-0.36
Composite foods	0.03	-0.06	0.25	0.20	0.15	0.25	0.27	0.69
Raw vegetables	-0.16	0.08	-0.13	0.67	0.47	0.00	-0.24	0.29
Vegetables with additional energy contain (peanut sauce, oil or coconut milk)/ salted vegetables	0.59	0.22	0.02	-0.11	0.61	0.19	0.13	0.08
Fruits	0.42	-0.01	0.08	0.35	0.82	-0.05	-0.03	0.12
Non-fried snacks	0.30	-0.06	0.14	0.33	0.27	0.19	0.10	0.82
Fried snacks/ fried side dishes	0.52	0.27	0.15	-0.17	0.31	0.07	0.53	-0.10
Desert sweet with coconut milk	0.52	0.42	-0.18	0.08	0.01	0.49	-0.08	0.20
Milk and milk product	0.11	-0.49	-0.06	0.43	0.00	-0.50	0.01	0.29
Tea, coffee, soft drink[††]	0.26	0.68	0.13	0.11	0.11	0.68	0.18	0.07

% of explained variance	11.7	10.2	8.8	7.7	13.9	10.7	8.8	7.3
% of cumulated variance	11.7	21.9	30.8	38.5	13.9	24.6	33.4	40.7

* Absolute values > 0.4 are underlined
VARIMAX
